# TRAIL inhibits oxidative stress in human aortic endothelial cells exposed to pro‐inflammatory stimuli

**DOI:** 10.14814/phy2.14612

**Published:** 2020-10-20

**Authors:** Hannah Forde, Emma Harper, Keith D. Rochfort, Robert G. Wallace, Colin Davenport, Diarmuid Smith, Philip M. Cummins

**Affiliations:** ^1^ Department of Endocrinology Beaumont Hospital and RCSI medical school Beaumont Dublin 9 Ireland; ^2^ School of Biotechnology Dublin City University Glasnevin Dublin 9 Ireland; ^3^ National Institute for Cellular Biotechnology Dublin City University Glasnevin Dublin 9 Ireland

**Keywords:** atherosclerosis, endothelium, oxidative stress, TRAIL

## Abstract

Studies suggest that tumor necrosis factor‐related apoptosis‐inducing ligand (TRAIL) has vasoprotective potential, as low levels of TRAIL cause accelerated vascular calcification, whereas exogenous TRAIL administration exhibits anti‐atherosclerotic activity. The mechanism of TRAIL‐mediated vasoprotection remains unclear. We studied the effects of TRAIL (100 ng/ml) on human aortic endothelial cells (HAECs) exposed to pro‐atherogenic conditions; (a) oscillatory shear stress (±10 dynes/cm^2^) using the ibidi µ‐slide fluidic system; (b) pro‐inflammatory injury, that is, tumor necrosis factor alpha (TNF‐α, 100 ng/ml) and hyperglycemia (30 mM d‐glucose). End‐points examined included inflammatory gene expression and reactive oxygen species (ROS) formation. TRAIL shifted the net gene expression toward an antioxidant phenotype in HAECs exposed to oscillatory shear stress. TRAIL significantly reduced ROS formation in HAECs exposed to both TNF‐α and hyperglycemia. Therefore, TRAIL appears to confer atheroprotective effects on the endothelium, at least in part, by reducing oxidative stress.

## Graphical abstract

Human aortic endothelial cells (HAECs) were treated with recombinant TRAIL in the presence and absence of pro‐inflammatory stimuli (Tumour necrosis factor alpha and hyperglycaemia). TRAIL reduced reactive oxygen species formation in HAECs exposed to pro‐inflammatory, pro‐atherogenic conditions.

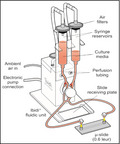

## 1 INTRODUCTION

Cardiovascular disease (CVD) is the leading cause of death in patients with type 2 diabetes mellitus (T2DM) and accounts for approximately 50% of deaths in this cohort (Leon & Maddox, 2015). The increased risk of cardiovascular death associated with diabetes has persisted, despite aggressive risk factor management and the availability of improved therapies in both diabetes and cardiovascular medicine. It is clear, therefore, that research should focus on developing new interventions to target early atherosclerotic plaque development, in an effort to reduce cardiovascular morbidity and mortality in this population.

Tumor necrosis factor‐related apoptosis‐inducing ligand (TRAIL) is a member of the tumor necrosis factor (TNF) superfamily (Wiley et al., 1995). It was previously thought that this protein was capable of inducing apoptosis in malignant cells only, with little or no effect on non‐malignant cells (Wiley et al., 1995). It is now clear, however, that TRAIL may have many roles through its interactions with five distinct receptors (Degli‐Esposti, 1999). TRAIL and its associated receptors exhibit a broad tissue distribution and all TRAIL receptors are expressed by the endothelium, justifying the endothelial cell responsiveness to this ligand (Zauli & Secchiero, 2006). Several in vivo studies have suggested that TRAIL exhibits vasoprotective properties in the vasculature (Di Bartolo et al., 2011; Secchiero et al., 2006). Indeed, Di Bartolo et al. (2011) and Secchiero et al. (2006) have demonstrated the ability of TRAIL to exert anti‐atherosclerotic and anti‐calcification effects, respectively, in mice.

There have also been numerous clinical studies examining serum levels of TRAIL in patient populations with high CVD burden, and among high‐risk patients, low levels of TRAIL are associated with a poorer prognosis (Secchiero et al., 2009; Volpato et al., 2011). Although these findings suggest that TRAIL may be protective against atherosclerosis, the exact mechanism of TRAIL‐mediated atheroprotection remains unclear. Preliminary data suggest that TRAIL may affect ROS expression within the endothelium. Zauli et al. (2003) demonstrated the ability of TRAIL to upregulate endothelial nitric oxide synthase (eNOS) in vascular endothelial cells leading to increased nitric oxide (NO) production, proposing an anti‐inflammatory, anti‐oxidant effect of TRAIL.

Using human aortic endothelial cells (HAECs), the primary objective of this study was to investigate the vasoprotective effects of TRAIL on the endothelium exposed to both hemodynamic (oscillatory shear stress, OSS) and humoral (TNF‐α, hyperglycemia) pro‐atherogenic stimuli.

## 2 MATERIALS AND METHODS

Unless otherwise stated, all reagents were purchased from Sigma‐Aldrich. HAECs and culture medium were purchased from PromoCell GmbH. Recombinant TRAIL was purchased from R&D Systems and TNF‐α was purchased from Merck Millipore. The ibidi µ‐slide flow system was utilized to conduct in vitro oscillatory flow experiments (Supporting Information). The Human Endothelial Cell Biology RT^2^ Profiler^™^ Array was purchased from Qiagen. Primers were purchased from Sigma Aldrich and Eurofins Genomics.

### 2.1 Cell culture

Human aortic endothelial cells were donated from a 45‐year‐old Caucasian female and had a population doubling time of 19 hr (catalog number C12271, Lot number 806130.1). Cells were cultured in an endothelial growth cell medium (catalog number C‐22020) containing the following supplements; fetal calf serum (0.05%), endothelial cell growth supplement (0.004 ml/ml), epidermal growth factor (10 ng/ml), heparin (90 µg/ml) and hydrocortisone (1 µg/ml). The media was also supplemented with penicillin (100 IU/ml) and streptomycin (100 µg/ml). Cells were seeded into P‐100 culture dishes (Sarstedt) and maintained in a humidified incubator at 37°C and 5% CO_2_/95% air. Cells were monitored by standard phase‐contrast microscopy (Nikon Eclipse TS100, Nikon) on a daily basis. For all experiments, cells were used between passages 8–13. Cells number and viability were routinely measured using the ADAM^™^ (Advanced Detection and Accurate Measurement) cell counter (Digital Bio) for seeding density purposes and data normalization.

For non‐flow experiments, HAECs were seeded into 6‐well culture dishes at a concentration of 200,000 cells per well. They were grown to confluency under static (non‐sheared) conditions and treated with recombinant TRAIL (100 ng/ml) in the presence and absence of pro‐atherogenic stimuli (TNF‐α 100 ng/ml, hyperglycemia 30 mM) for 24 hr. The control population of cells was those untreated cells that were not exposed to TRAIL/TNF‐α/hyperglycemia. Post‐treatment, cells were stained with dihydroethidium (DHE) and monitored for ROS production by flow cytometry. All samples were stored at −80°C and assayed within 3 months.

### 2.2 Gel electrophoresis

Extraction of total ribonucleic acid (RNA) was achieved using the TRIzol^™^ RNA extraction protocol (Thermo Fisher Scientific) and the Applied Biosystems^™^ complementary deoxyribonucleic acid (cDNA) reverse transcription kit (Thermo Fisher Scientific), respectively. Prior to reverse transcription, RNA samples underwent purification with a DNAse treatment kit (Sigma‐Aldrich). Primers for death receptor 4 (DR4), death receptor 5 (DR5), decoy receptor 1 (DcR1), and decoy receptor 2 (DcR2) were generated against sequences identified in the nucleotide database provided online by the National Center for Biotechnology Information (NCBI). A master mix containing 1 µl of sample cDNA (500 ng), 2.5 µl of reaction buffer (10×), 2 µl of deoxynucleotide triphosphate (dNTP; 10 mM), 1.5 µl of magnesium chloride (MgCl_2_; 25 mM), 15.75 µl of nuclease‐free water, and 0.125 µl of Taq polymerase was prepared. One microliter of forward primer and 1 µl of reverse primer were added to the mix, which was then placed in a thermal cycler to allow deoxyribonucleic acid (DNA) amplification. The DNA products of the various primer sets were then analyzed via 1% agarose gel electrophoresis. Gels were visualized with the transilluminator setting on a G‐Box (Syngene) to confirm predicted band sizes for each primer pair. The sequences for the primers are as follows; DR4 (198 bp): Forward: 5ʹ‐AAGAGAGAAGTCCCTGCAC‐3ʹ; Reverse: 5ʹ‐TCACAACCAAAATCACCC‐3ʹ; DR5 (506 bp): Forward: 5ʹ‐GCCTCATGGACAATGAGATAAAGGTGGCT‐3ʹ; Reverse: 5ʹ‐CCAAATCTCAAAGTACGCACAAACGG‐3ʹ; DcR1 (611 bp): Forward: 5ʹ‐GAAGAATTTGGTGCCAATGCCACTG‐3ʹ; Reverse: 5ʹ‐CTCTTGGACTTGGCTGGGAGATGTG‐3ʹ; Dcr2 (181 bp): Forward:5ʹ‐TTACACCATTGCTTCCAAC‐3ʹ; Reverse: 5ʹ‐TACTGACCTTGACCATCCC‐3ʹ.

### 2.3 Ibidi cell experiments; oscillatory flow ± TRAIL

The ibidi system is a novel, state‐of‐the‐art culture model of pro‐atherogenic OSS known to promote atheroma formation (Davies, 2009). The system consists of a pump, two fluidic units, two perfusion sets (tubing), and a 0.6 mm flow µ‐slide (a chambered coverslip; Ibidi, Germany, catalog number 80186; Figure S1). Prior to exposure, HAECs were seeded onto a flow µ‐slide, at a concentration of 200,000 cells per slide and transferred to an Olaf humidifying chamber (catalog. number 80008) to ensure sealed incubation in a constant atmosphere, and placed in the incubator for 24 hr where they grew to confluency. Culture media (100 ng/ml of TRAIL) was added to the 10 ml syringe reservoirs (catalog number 10971) 24 hr later. The µ‐slide was attached to a fluidic unit and each unit was placed in a laminar flow cabinet and connected to an air pressure pump, controlled by the PumpControl software program on a dedicated ibidi‐laptop. The pump was set to expose confluent HAECs to OSS levels of ±10 dynes/cm^2^ for 24 hr. Post‐treatment, messenger RNA (mRNA) was extracted from the cells for analysis.

### 2.4 Polymerase chain reaction (PCR) microarray

The Human Endothelial Cell Biology RT^2^ Profiler^™^ PCR Array (Qiagen) was utilized to screen for gene targets by TRAIL (Table S1). Total RNA was harvested from ibidi samples using the mirVana® Isolation kit. Reverse transcription was performed using an RT^2^ PreAMP cDNA Synthesis kit (Qiagen). CDNA was pre‐amplified with RT^2^ PreAMP Pathway Primer mix (Qiagen). PCR reaction mixtures were as follows: 1,350 µl of 2× RT^2^ SYBR Green Master mix (Qiagen), 102 µl of cDNA synthesis reaction, and 1,248 µl of RNase‐free water. A 25 µl of aliquot was added to each well, sealed and centrifuged at 1,000*g* for 1 min. Amplification of cDNA targets was achieved using the Applied Biosystems 7900HT Fast RT‐PCR system. Samples were denatured at 95°C for 10 min, followed by 40 cycles at 95°C for 15 s and 60°C for 1 min. Cyclic threshold (C_T_) values were calculated for each reaction and results were analyzed using a web‐based software (www.SABiosciences.com/pcrarraydataanalysis.php).

### 2.5 Flow cytometry

ROS levels were quantified using flow cytometry with dihydroethidium (DHE) staining. Confluent HAECs were labeled with 3 µM DHE (Sigma Aldrich) 30 min prior to the completion of the 24‐hr stimulus exposure time. Post‐treatment, HAECs were trypsinized from 6‐well plates and centrifuged for 6 min at 0.1*g* at room temperature. The pellet was washed in fluorescence‐activated cell sorting (FACS) buffer (filtered phosphate‐buffered saline (PBS) containing 2% fetal bovine serum and 0.1% sodium azide) and centrifuged again for 6 min at 0.1*g*. Cells were then resuspended in 500 µl of FACS buffer and read for 10,000 events with the BD FACS Aria (BD Biosciences). FlowJo (v 10.0) software was employed to analyze all flow cytometry data.

### 2.6 Statistical analysis

Statistical analysis was performed using a statistical software package (SPSS version 23.0). Data from the in vitro experiments were expressed as *M* ± *SEM*. Unless otherwise indicated, all experiments were conducted on three separate occasions (*n* = 3). Statistical comparisons between control versus treated groups were tested using Students *t* test/Analysis of variance. A *p* value of ≤.05 was considered significant. Holm–Bonferroni test was used in post hoc analysis to control for multiple comparisons.

## 3 RESULTS

### 3.1 TRAIL receptor expression by HAECs

**Figure 1:**
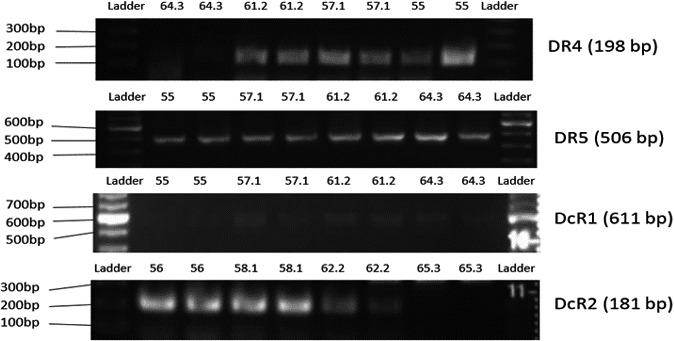
TRAIL receptor expression by untreated HAECs. Each lane represents the PCR product derived at a specific primer melting temperature. Primer melting temperatures ranged from 55 to 66°C and samples were loaded in duplicate. bp, base; DcR1, decoy receptor 1; DcR2, decoy receptor 2; DR4, death receptor 4; DR5, death receptor 5; HAECs, human aortic endothelial cells; TRAIL, tumor necrosis factor‐related apoptosis‐inducing ligand

Following primer generation, the expression of membrane‐bound TRAIL receptors, by HAECs under basal conditions was examined using PCR and agarose gel electrophoresis. HAECs were found to express the four TRAIL receptors, with DcR1 appearing to be expressed at lower levels compared with DR4, DR5, and DcR2 (Figure 1). DR5 appeared to be the most abundantly expressed receptor in this cell population. DR4 and DcR2 were expressed but to a lesser extent than DR5.

### 3.2 Effect of TRAIL on gene expression in HAECs exposed to OSS

To investigate the effect of TRAIL on endothelial gene expression under pro‐atherogenic conditions, an in vitro oscillatory flow model (±10 dynes/cm^2^, 24 hr) was used in conjunction with RT‐qPCR microarray profiling to investigate potential target genes that may be upregulated or downregulated by TRAIL (100 ng/ml, 24 hr) during atherogenic flow‐mediated injury. Gene expression by TRAIL‐treated HAECs exposed to OSS was compared to that induced by HAECs exposed to OSS in the absence of TRAIL (i.e., control). Under oscillatory flow, TRAIL upregulated superoxide dismutase 1 (SOD1), heme‐oxygenase 1 (HMOX1), and nitric oxide synthase 3 (NOS3); genes that encode enzymes with antioxidant properties (Table 1). Furthermore, TRAIL upregulated calcitonin‐related polypeptide alpha (CALCA) which encodes the vasoprotective calcitonin gene‐related peptide (CGRP). TRAIL also downregulated the pro‐oxidant endothelin 1 (EDN1), chemokine ligand 2 (CCL2), caveolin 1 (CAV1), and transforming growth factor beta (TGF‐β). Thus, under OSS, TRAIL appears to shift gene expression toward a more antioxidant phenotype.

**Table 1:** Genes up/downregulated by tumor necrosis factor‐related apoptosis‐inducing ligand (TRAIL) (100 ng/ml) in HAECs exposed to OSS (±10 dynes/cm^2^) for 24 hr. *N* = 3. Results pooled for display purposes

Gene	Description	Fold change	Gene	Description	Fold change	Gene	Description	Fold change	Gene	Description	Fold change	Gene	Description	Fold change
ACE	Angiotensin I converting enzyme 1	1.52		CASP8 and FADD‐like apoptosis regulator	−0.56	HIF1A	Hypoxia inducible factor 1, alpha subunit	−0.88	MMP9	Matrix metallopeptidase 9	5.2	SERPINE1	Serpin peptidase inhibitor, clade E	1.38
ADAM17	ADAM metallopeptidase domain 17	2.27	COL18A1	Collagen, type XVIII, alpha 1	1.97	HMOX1	Heme oxygenase 1	3.71	NOS3	Nitric oxide synthase 3	2.0	SOD1	Superoxide dismutase 1, soluble	1.4
ANGPT1	Angiopoietin 1	−0.97	CX3CL1	Chemokine (C‐X3‐C motif) ligand 1	1.42	ICAM1	Intercellular adhesion molecule 1	3.17	NPR1	Natriuretic peptide receptor A	2.47	SPHK1	Sphingosine kinase 1	1.44
ANXA5	Annexin A5	1.17	EDN1	Endothelin 1	−0.27	IL11	Interleukin 11	−0.2	OCLN	Occludin	5.6	TEK	TEK tyrosine kinase, endothelial	2.64
BAX	BCL2‐associated X protein	−0.82	EDN2	Endothelin 2	−0.51	IL3	Interleukin 3	4.27	PDGFRA	Platelet‐derived growth factor receptor, alpha polypeptide	−0.32	TFPI	Tissue factor pathway inhibitor	−0.64
BCL2	B‐cell CLL/lymphoma 2	−0.48	EDNRA	Endothelin receptor type A	4.66	IL6	Interleukin 6 (interferon, beta 2)	1.68	PECAM1	Platelet/endothelial cell adhesion molecule	3.36	TGFB1	Transforming growth factor, beta 1	−0.84
BCL2L1	BCL2‐like 1	1.53	ENG	Endoglin	3.41	ITGA5	Integrin, alpha 5	2.14	PF4	Platelet factor 4	−0.79	THBD	Thrombomodulin	2.17
CALCA	Calcitonin‐related polypeptide alpha	15.5	F2R	Coagulation factor II (thrombin) receptor	1.2	ITGAV	Integrin, alpha V	1.02	PGF	Placental growth factor	−0.62	THBS1	Thrombospondin 1	−0.72
CASP1	Caspase 1, apoptosis‐related cysteine peptidase	1.79	F3	Coagulation factor III (thromboplastin, tissue factor)	107.5	ITGB1	Integrin, beta 1	1.5	PLAU	Plasminogen activator, urokinase	−0.57	TIMP1	TIMP metallopeptidase inhibitor 1	−0.84
CASP3	Caspase 3, apoptosis‐related cysteine peptidase	−0.76	FAS	Fas (TNF receptor superfamily, member 6)	5.39	ITGB3	Integrin, beta 3	1.57	PTGIS	Prostaglandin I2 (prostacyclin) synthase	6.68	TNF	Tumor necrosis factor	4.29
CAV1	Caveolin 1, caveolae protein, 22 kDa	−0.68	FGF1	Fibroblast growth factor 1 (acidic)	−0.43	KDR	Kinase insert domain receptor	2.13	PTGS2	Prostaglandin‐endoperoxide synthase 2	−0.94	TNFSF10	Tumor necrosis factor (ligand) superfamily, member 10	2.13
CCL2	Chemokine (C‐C motif) ligand 2	−0.99	FGF2	Fibroblast growth factor 2 (basic)	−0.61	KIT	V‐kit Hardy‐Zuckerman 4 feline sarcoma viral oncogene homolog	−0.09	PTK2	Protein tyrosine kinase 2	1.09	TYMP	Thymidine phosphorylase	−0.59
CCL5	Chemokine (C‐C motif) ligand 5	−0.69	FLT1	Fms‐related tyrosine kinase 1	1.24	MMP1	Matrix metallopeptidase 1	1.93	SELE	Selectin E	4.62	VCAM1	Vascular cell adhesion molecule 1	5.23
CDH5	Cadherin 5, type 2 (vascular endothelium)	1.78	FN1	Fibronectin 1	1.48	MMP2	Matrix metallopeptidase 2	1.48	SELL	Selectin L	16.3	VEGFA	Vascular endothelial growth factor A	2.1
									SELPLG	Selectin P ligand	2.13	VWF	Von Willebrand factor	6.9

### 3.3 Effect of TRAIL on ROS generation by HAECs exposed to pro‐inflammatory conditions

**Figure 2:**
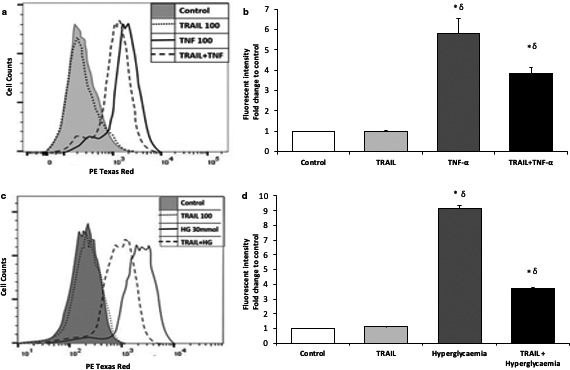
TRAILs effect on pro‐inflammatory cytokine‐induced ROS release by HAECs. (a + b) TNF‐ α exposure. (a) Flow cytometry overlay of DHE fluorescence intensity. (b) Histogram of mean DHE fluorescence relative to control for each condition outlined. (c + d) Hyperglycemia exposure. (c) Flow cytometry overlay of DHE fluorescence intensity. (d) Histogram of mean DHE fluorescence relative to control for each condition outlined *N* = 3, Data are presented as mean ± *SEM*. *=*p* < .05 compared to control. σ =*p* < .05 compared to TRAIL. DHE, dihydroethidium; HAECs, human aortic endothelial cells; HAECs, human aortic endothelial cells; ROS, reactive oxygen species; *SEM*, standard error of the mean; TNF‐α, tumor necrosis factor alpha; TRAIL, tumor necrosis factor‐related apoptosis‐inducing ligand

As our preliminary study suggested that TRAIL may promote the activation of antioxidant gene pathways, the effect of TRAIL on TNF‐α‐induced oxidative stress in static HAECs was quantitatively assessed by flow cytometry. TRAIL (100 ng/ml, 24 hr) alone had no effect on basal ROS levels whereas the treatment of HAECs with TNF‐α (100 ng/ml, 24 hr) significantly increased ROS formation. TRAIL, however, significantly attenuated TNF‐α‐induced ROS generation by 35%, thus demonstrating an antioxidant effect (Figure 2a+b). This experiment was repeated using hyperglycemia‐induced ROS formation as the injurious stimulus and a similar pattern was observed. The hyperglycaemic stimulus (30 mmol, 24 hr) significantly increased ROS production, whilst TRAIL (100 ng/ml, 24 hr) alone had no effect. In combination, however, TRAIL significantly attenuated ROS generation by 60% in HAECs exposed to hyperglycaemic conditions, providing further evidence of an antioxidant effect of TRAIL (Figure 2c+d).

## 4 DISCUSSION

A growing body of evidence points to cardioprotective effects and therapeutic benefits of TRAIL. In vivo studies have demonstrated that TRAIL^‒/‒^ mice develop accelerated atherosclerosis with features of plaque instability (Di Bartolo et al., 2011). Moreover, atherosclerosis is ameliorated by systemic TRAIL delivery in apoE^‒/‒^ mice (Secchiero et al., 2006). The pathways activated by TRAIL to confer atheroprotection within the vasculature are not well understood, however. This study aimed to provide mechanistic insight into the nature of TRAIL's vasoprotective properties.

We illustrated the transmembrane receptors expressed by this population of HAECs and demonstrated mRNA expression of DR4, DR5, DcR1, and DcR2 in these cells. This is consistent with the literature. Li et al. (2003) reported mRNA expression of DR4, DR5, and DcR2 expression in human umbilical vein endothelial cells (HUVECs), whilst Secchiero et al. (2003) confirmed protein expression of DR4, DR5, DcR1, and DcR2 in both HAECs and HUVECs by flow cytometry. Consistent with our observations, DR5 was more abundantly expressed than the other receptors (Secchiero et al., 2003). TRAIL does have the potential to bind with a fifth decoy receptor; osteoprotegerin. However, HAECs only express small amounts of OPG in basal conditions, and OPG itself has low affinity for TRAIL (Davenport et al., 2018; Wang & El‐Deiry, 2003).

After demonstrating TRAIL receptor expression, we conducted preliminary studies to determine the effect of TRAIL on gene expression in HAECs exposed to the pro‐atherogenic stimulus, OSS. Hemodynamic forces play an important role in atherosclerosis and areas exposed to low and oscillating shear stress such as branch points and bifurcations, are predisposed to the development of atherosclerotic plaque (Moore et al., 1994). Previous flow studies have shown that disturbed blood flow patterns upregulate a number of genes implicated in atherosclerosis such as vascular cell adhesion molecule 1 (VCAM1), E‐selectin, and monocyte chemoattractant protein 1 (MCP1) (Brooks et al., 2002). For this study, the ibidi flow system was utilized to model oscillatory flow in vitro in the presence and absence of TRAIL, and a PCR microarray profiling a panel of endothelium biology‐specific genes was used to determine potential targets for TRAIL. TRAIL upregulated a number of antioxidant and anti‐inflammatory genes including SOD1, HMOX1, NOS3, and CALCA. SOD1 is a vasoprotective enzyme which ameliorates atherosclerosis in the aortae of SOD1 transgenic mice (Tribble et al., 1999). SOD1 deficiency is also associated with increased vascular superoxide levels and endothelial dysfunction (Didion et al., 2002). HMOX1 is another enzyme with vasoprotective properties that is upregulated in response to oxidative stress and inflammatory stimuli (True et al., 2007). In apoE deficient mice, HMOX1 also attenuates atherosclerotic plaque formation (Juan et al., 2001). NOS3 is responsible for generating NO, which has many anti‐atherosclerotic effects (Cheang et al., 2011). Finally, CALCA displays vasoprotective properties by inhibiting macrophage infiltration and the expression of inflammatory mediators, including TNF‐α (Zhang et al., 2010).

The upregulation of antioxidant genes by TRAIL was coupled with the downregulation of EDN1; a pro‐oxidant gene that is also downregulated by atheroprotective laminar flow (Brooks et al., 2002). TRAIL reduced mRNA levels of CCL2, CAV1, and TGF‐β. These genes encode proteins that have been associated with increased oxidative stress in various cell types (Carmona‐Cuenca et al., 2006; Dasari et al., 2006; Hackel et al., 2013). Hence, in HAECs exposed to pro‐atherogenic OSS, TRAIL appears to switch the net gene expression toward an antioxidant phenotype. This led us to hypothesize that TRAIL exerts atheroprotective effects in endothelial cells, by reducing oxidative stress.

As the findings from the microarray study suggested an antioxidant effect of TRAIL in response to pro‐inflammatory OSS, we investigated the effect of TRAIL on ROS formation in HAECs exposed to pro‐inflammatory cytokine‐induced injury. We demonstrated that TRAIL significantly attenuated TNF‐α‐ and hyperglycemia‐induced ROS production. The ability of TRAIL to reduce oxidative stress in the vasculature is a relatively novel finding, with only two groups reporting similar results to date. Liu et al. (2014) demonstrated an improvement in endothelial function in response to TRAIL treatment in streptozotocin‐induced diabetic rats. This group confirmed these vasoprotective properties by demonstrating TRAILs ability to attenuate hyperglycemia‐induced intracellular ROS production in vitro (Liu et al., 2014). Similarly, Cholan et al. (2018) reported endothelial dysfunction in TRAIL‐deficient mice and corroborated these findings in vitro by demonstrating the inhibition of angiotensin II‐induced ROS generation by TRAIL in endothelial cells.

While our findings, in conjunction with the aforementioned studies, provide evidence for an antioxidant effect of TRAIL, other groups have reported conflicting results. Li et al. (2013) demonstrated the induction of oxidative stress and endothelial dysfunction in coronary artery endothelial cells by TRAIL, through membrane raft clustering and the activation of the potent pro‐oxidant nicotinamide adenine dinucleotide phosphate (NADPH) oxidase enzyme gp91phox subunit. Another group also observed increased oxidative stress in lymphocytes and splenocytes treated with TRAIL (Dumitru & Gulbins, 2006).

Why TRAIL demonstrates antioxidant activity in some studies and pro‐oxidant activity in others is not clear. It is likely that the mechanism of TRAIL‐mediated vasoprotection is dependent upon whether or not the cells are in an injurious state as well as the type of injury sustained (OSS vs. inflammatory cytokine‐induced injury). There are different types of oxidative injury (biomechanical and humoral) and the defense mechanisms and pro‐survival strategies mounted by cells to adapt to stress, are determined by the type and level of the cellular insult (Fulda et al., 2010).

It is noteworthy that the aforementioned in vitro studies were conducted on static, non‐sheared cells. Whilst it is difficult to replicate an environment in vitro that mimics the complex pathophysiological processes involved in atherosclerosis, models incorporating controllable flow are thought to be the best use of in vitro systems for atherosclerosis research (Ohayon et al., 2020). Our unique approach using the ibidi µ‐slide pump system enabled us to precisely control flow conditions to produce physiological wall shear stress on the cultured HAECs. To the best of our knowledge, this is the first study to examine the effect of TRAIL on HAECs under flow conditions.

In summary, this study adds to the existing body of evidence that TRAIL is a pleiotropic molecule with vasoprotective and atheroprotective properties. Through these experiments, we have garnered an improved understanding of the effects of TRAIL on various genes involved in redox signaling. Our study suggests that TRAIL significantly reduces oxidative stress in the vasculature during pro‐atherogenic conditions. The effect of TRAIL on redox signaling warrants further investigation so that its therapeutic potential can be realized.

## CONFLICT OF INTERESTS

The authors declare that there are no conflicts of interest regarding the publication of this paper.

## AUTHOR CONTRIBUTIONS

HF conducted the experiments assisted by EH, KR, and RW; EH carried out the western blots; HF interpreted the results and drafted the manuscript; EH, KR, RW, CD, DS, and PC contributed to the preparation of the manuscript; HF, KR, DS, and PC conceived the study and participated in its design. All authors have read and approved the final manuscript.

## Supporting information



Supplementary Material
