# Supplementary material for: TRAIL inhibits oxidative stress in human aortic endothelial cells exposed to pro‐inflammatory stimuli
Source: Physiol Rep. 2020 Oct 20;8(20):e14612. doi: 10.14814/phy2.14612 (PMC7575224; doi:10.14814/phy2.14612)
Supplement: Supplementary file 1 — Supplementary Material [file PHY2-8-e14612-s001.docx]

**Supplementary material**

Table S1. Gene list for Endothelial Cell Biology, Gene array (Qiagen)

| **Gene Abbreviation** | **Gene name** |
| --- | --- |
| ACE | Angiotensin converting enzyme |
| ADAM17 | ADAM Metallopeptidase domain 17 |
| AGT | Angiotensinogen |
| AGTR1 | Angiotensin II receptor 1 |
| ALOX5 | Arachidonate 5-lipoxygenase |
| ANGPT1 | Angiopoietin 1 |
| ANXA5 | Annexin 5 |
| APOE | Apolipoprotein E |
| BAX | BCL-2-associated X protein |
| BCL2 | B-cell lymphoma 2 |
| BCL2L1 | BCL-2-like protein 1 |
| CALCA | Calcitonin related peptide alpha |
| CASP1 | Caspase 1 |
| CAV1 | Caveolin 1 |
| CCL2 | Chemokine ligand 2 |
| CCL5 | Chemokine ligand 5 |
| CDH5 | Cadherin 5 |
| CFLAR | CASP8 and FADD-like apoptosis regulator |
| COL18A1 | Collagen Type XVIII Alpha 1 |
| COX2 | Cyclooxygenase 2 |
| CX3CL1 | Chemokine ligand 1 |
| EDN1 | Endothelin 1 |
| EDNRA | Endothelin receptor A |
| ENG | Endoglin |
| FAS | Fas cell surface death receptor |
| FASLG | Fas ligand |
| F3 | Thromboplastin tissue factor |
| FGF1 | Fibroblast growth factor 1 |
| FGF2 | Fibroblast growth factor 2 |
| FLT1 | Fms-related Tyrosine Kinase 1 |
| FN1 | Fibronectin 1 |
| F2R | Thrombin receptor |
| HIF1A | Hypoxia Inducible Factor 1A |
| HMOX1 | Heme oxygenase 1 |
| ICAM | Intracellular cellular adhesion molecule |
| IL1B | Interleukin 1B |
| IL3 | Interleukin 3 |
| IL6 | Interleukin 6 |
| IL7 | Interleukin 7 |
| IL11 | Interleukin 11 |
| ITGA5 | Integrin Alpha |
| ITGAV | Integrin Alpha V |
| ITGB1 | Integrin Beta 1 |
| ITGB3 | Integrin Beta 3 |
| eNOS | Endothelial nitric oxide synthase |
| KDR | Kinase Insert Domain receptor |
| KIT | v-Kit Hardy Zuckerman |
| KLK3 | Kallikrein related peptidase 3 |
| MMP1 | Matrix metalloproteinase 1 |
| MMP2 | Matrix metalloproteinase 2 |
| MMP9 | Matrix metalloproteinase 9 |
| NPPB | Natriuretic peptide B |
| NPR1 | Natriuretic peptide receptor 1 |
| OCLN | Occludin |
| PDGFRA | Platelet derived growth factor receptor alpha |
| PECAM1 | Platelet endothelial cell adhesion molecule |
| PF4 | Platelet factor 4 |
| PGF | Placental growth factor |
| PLAT | Plasminogen activator, tissue |
| PLAU | Plasminogen activator, urokinase |
| PLG | Plasminogen |
| PROCR | Protein C receptor |
| PTGIS | Prostacyclin Synthase |
| PTK2 | Protein Tyrosine Kinase 2 |
| SELE | E-Selectin |
| SELL | L-Selectin |
| SELPLG | P-Selectin ligand |
| SERPINE1 | Serpin Peptidase Inhibitor 1 |
| SOD1 | Superoxide dismutase 1 |
| SPHK1 | Sphingosine kinase 1 |
| TEK | Tyrosine Kinase |
| TFP1 | Transferrin pseudogene 1 |
| TGFβ | Transforming growth factor Beta |
| THBD | Thrombomodulin |
| THBS1 | Thrombospondin 1 |
| TIMP1 | Tissue Inhibitor of Metalloproteinases 1 |
| TNF | Tumour necrosis factor |
| TRAIL | Tnf related apoptosis inducing ligand |
| TYMP | Thymidine Phosphorylase |
| VCAM | Vascular cellular adhesion molecules |
| VEGFA | Vascular endothelial growth factor A |
| VWF | Von Willebrand Factor |


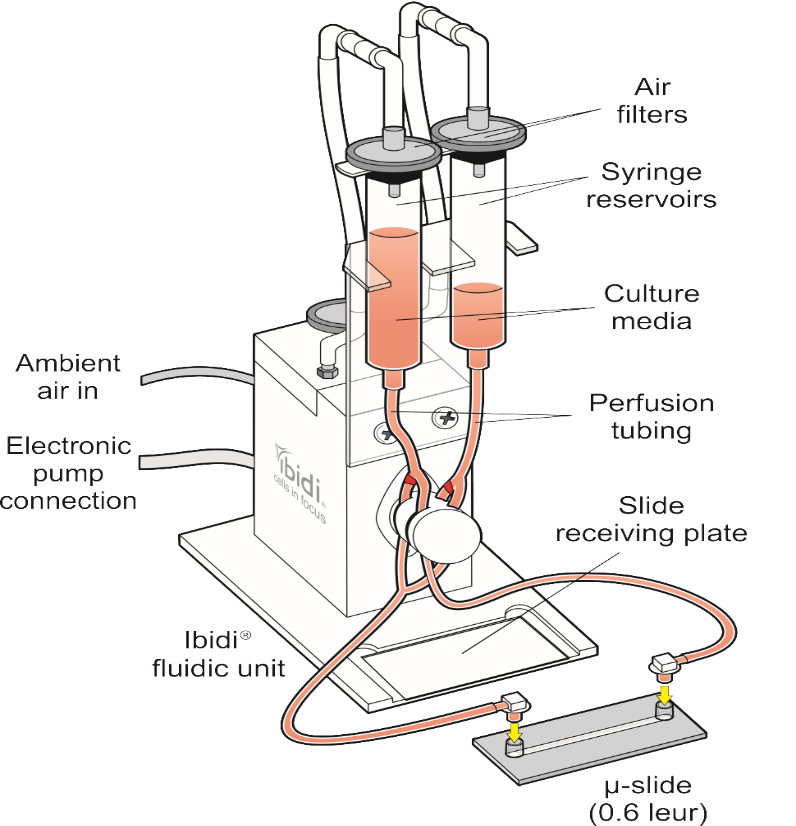


**Figure S1. Structure of a fluidic unit in an IBIDI µ-slide pump system**. Media is contained in the syringe reservoirs. The flow of media across the µ-slide is determined by the settings on the air pressure pump which is connected to the fluidic unit. Image used with permission from Mr. Robert Wallace.
